# The importance of multi‐year studies and commercial yield metrics in measuring pollinator dependence ratios: A case study in UK raspberries *Rubus idaeus* L.

**DOI:** 10.1002/ece3.10044

**Published:** 2023-05-07

**Authors:** Imogen C. Ryan, Lynn V. Dicks, Jack D. Shutt

**Affiliations:** ^1^ School of Biological Sciences University of East Anglia Norwich UK; ^2^ Department of Zoology University of Cambridge Cambridge UK

**Keywords:** exclusion, fruit set, fruit weight, marketable quality threshold, pollination, soft fruit

## Abstract

The benefit of pollinators to crop production is normally calculated using “pollinator dependence ratios,” which reflect the proportion of yield lost (here reported as a value between 0 and 1) in the absence of pollinators; these ratios are quantified experimentally using pollinator exclusion experiments. Pollinator dependence ratio estimates can vary considerably for a single crop, creating large, frequently overlooked, uncertainty in economic valuations of pollinators. The source of this variation is usually unclear. We experimentally measured the pollinator dependence ratio of two UK commercial cultivars of raspberry *Rubus idaeus* L*.*, using a range of yield metrics—fruit set, marketable fruit set, fruit weight, and marketable fruit weight—over 3 years (2019–2021), to quantify the effects of yield metric, interannual variation, and cultivar on pollinator dependence ratio estimates. We found a difference in the pollinator dependence ratio for fruit set of 0.71 between 2019 and 2020, showing the importance of carrying out exclusion studies over multiple years. Averaged over multiple years and two cultivars, the dependence ratio was 0.68 measured using marketable fruit set and 0.64 using marketable fruit weight. Imposing a quality threshold (size and shape) below which fruits would not be of commercial value (marketable fruit set/weight) dramatically increased both the pollinator dependence ratio and subsequent economic valuations of pollination service derived from it. Our study shows that, for raspberry, estimates of the pollinator dependence ratio, and therefore, the economic value of insect pollinators, are highly sensitive to the choice of yield metric and can change between years and cultivars. Many economic decisions about pollinator management, at farm, regional and national scales rely on estimates of pollinator dependence. We, therefore, recommend that for estimating pollinator dependence ratios, pollinator exclusion studies are conducted over three or more years and use yield metrics that incorporate quality criteria linked to actual market values and commercial thresholds.

## Graphical abstract

Pollinator dependence ratios are important for quantifying the benefit of pollinators to crops. Pollinator dependence ratios often are not calculated using commercially relevant yield metrics and are frequently informed by single‐year studies. We show that the pollinator dependence on raspberry varied greatly between years and with differing yield metrics. Imposing a threshold fruit quality (size and shape) below which fruits would not be of commercial value (marketable fruit set) dramatically increased both the pollinator dependence ratio and subsequent economic valuations of pollination service derived from it. Our results show the influence of yield metrics on pollinator dependence estimates and the economic value of pollinators, and we recommend that pollinator exclusion studies are conducted over multiple years (three or more) and use yield metrics that incorporate quality criteria linked to actual market values and commercial thresholds of the product in question.

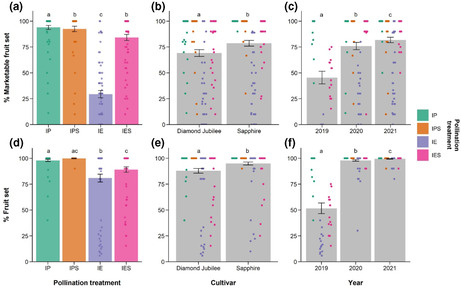

## 1 INTRODUCTION

It is well known that pollinators are important for the reproduction of flowering plants. An estimated 79% of angiosperms have improved seed sets in the presence of pollinators (Rodger et al., 2021), whereas 75% of the world's major food crops depend on pollination to some extent, to produce the edible or marketable parts of the plant (Klein et al., 2007). This dependence translates to between 5% and 8% (by volume) of human food produced globally being a direct result of animal pollination (Aizen et al., 2009; Potts et al., 2016). These estimates are all based on empirically derived dependence ratios, which measure the loss of yield in the absence of pollinators (Gallai et al., 2009), for each type of crop. The dependence ratios from Klein et al. (2007), along with 2009 market prices and production, were used by Lautenbach et al. (2012) to provide an annual economic value of pollinators to global crop output. Inflated to 2015 US$, this equates to US$235–577 billion per year (Potts et al., 2016).

These estimates of the overall economic value of pollinators for crop production allow us to assess the potential economic consequences of observed pollinator declines (e.g., UK declines shown by Powney et al., 2019). They are used as motives or incentives for pollinator conservation (Kleijn et al., 2015), and to evaluate how much should reasonably be invested in crop pollination service management (Allsopp et al., 2008) and pollinator monitoring (Breeze et al., 2021).

Exclusion studies can be used to measure dependence ratios by preventing pollinators from visiting study flowers using mesh bags or cages and comparing the resulting yield to that from flowers that had access to pollinators. Pollination deficits (any loss in yield due to a lack of pollination) can be quantified by providing additional pollen to flowers that have access to pollinators (usually done by hand using a paintbrush) and comparing the resulting yield to flowers that were not pollen supplemented. Pollination deficits can be used to determine whether and how much pollination service management needs to be improved to maximize yields. Pollinator exclusion and pollen supplementation experiments have been used to quantify the pollinator dependence and pollination deficits of over 80 different crops (Klein et al., 2007), including apple (Garratt et al., 2014), strawberry (Klatt et al., 2014), and raspberry (Andrikopoulos & Cane, 2018; Cane, 2005; Chagnon et al., 1991; Chen et al., 2021; Ellis et al., 2017; Prodorutti & Frilli, 2008). Pollinator dependence and pollen limitation have also been assessed for some wild flowering plants (Koch et al., 2020; Ratto et al., 2018; Rodger et al., 2021).

The yield metrics used for comparison between pollination treatments, and thus the basis for dependence and economic valuations, vary between studies. Even within yield quantity parameters, for example, mass per unit or the number of units, selecting a yield parameter of “direct economic benefit” is important as the relationships between yield metrics can be nonlinear as shown in field bean by Bishop et al. (2020). Yield metrics used for fruit crops include the proportion of crop flowers that produce fruits (% fruit set) and the weight and size of the resulting fruit. These metrics reflect commercial value, as produce is normally paid for by weight or volume. However, in industry, there can be minimum thresholds for commercially acceptable sizes or weights, below which fruits cannot be sold or have reduced value. These thresholds are frequently overlooked. Many raspberry pollination studies, for example, do not account for a lower size threshold of marketability in measuring yield attributable to insect pollinators (Andrikopoulos and Cane 2018; Cane 2005; Chagnon et al., 1991; Chen et al., 2021, 2022; Prodorutti and Frilli 2008). Species‐specific yield metrics used to reveal details of the pollination process, such as drupelet set in raspberries (Andrikopoulos & Cane, 2018), can also be less relevant to the industry.

As well as influencing the *quantity* of fruit, insect pollination can also impact esthetic fruit qualities such as shape and color. These metrics, along with size or weight, can affect the price per tonne, for example, when produce is given a classification or grading for the market, based on both quantity and quality metrics as for apples (Garratt et al., 2014). Accounting for the difference in price per unit between classes is therefore important for economic valuations of pollinators to crops as shown by Garratt et al. (2014). These esthetic qualities, such as shape, color, or, in the case of raspberries, uniformity and wholeness can also result in fruit being classified as unmarketable and not entering the marketplace at all (BerryWorld pers. Comms, December 2019). It is therefore important to measure the benefits to crop quality as well as quantity when calculating the contribution of pollinators to crop production value. This is especially true for those species, such as strawberries, dessert apples, and raspberries, which are self‐fertile in their reproduction and able to produce some fruit in the absence of pollinators but require pollinators to produce marketable quality fruit, as measured by Ellis et al. (2017), Garratt et al. (2014), and Klatt et al. (2014). Yield quality is also important for seed crops as their market value lies in the viability and vigor of the seeds and so including seed quality in pollinator dependence valuations, such as in Fijen et al. (2018), is essential for measuring the true value of pollinators to crop production. Including fruit that is too small or light, or otherwise unsuitable for the market in calculations of pollinator dependence ratios and resulting economic valuations will underestimate the economic value of pollinators to food crops in cases where fruit quality, or price band, is improved by pollination. Likewise, the benefit of pollinators to yield can be overestimated if quality metrics are not considered where high densities of pollinators have detrimental impacts on fruit quality (Aizen et al., 2014; Monasterolo et al., 2022; Sáez et al., 2014).

In 2020, UK raspberry (*Rubus idaeus* L.) production was valued at £133.3 million, 12.8% of total UK fruit production value for that year and the third largest income for a single type of fruit behind strawberries and dessert apples (Defra, 2021). Despite most commercial cultivars being self‐fertile, that is, being able to produce seeds with pollen from the same plant, unlike wild varieties, the structure of the reproductive parts of *Rubus idaeus* flowers prevents unaided complete self‐pollination (Free, 1970; McGregor, 1976) and thus the production of commercially viable fruit. Each pollinated pistil produces a single fruiting body, called a drupelet, containing a single seed, or in some cases two (Funt & Hall, 2013). The more stigmas that are pollinated, the more drupelets will develop and the larger the fruit. Raspberries are termed an aggregate fruit, as each “fruit”, or “berry”, is made up of multiple drupelets. When picked they are separated from their receptacle. This means that to remain whole, there needs to be enough cohesion between drupelets. If too few drupelets develop, the fruit is crumbly and unmarketable (Andrikopoulos & Cane, 2018). The majority of a flower's ovules need to be fertilized to produce commercially marketable fruit (Cane, 2005). The benefit of pollinators to fruit quality can therefore make the difference between marketable and unmarketable fruit.

Various pollinator exclusion studies (Andrikopoulos & Cane, 2018; Cane, 2005; Chagnon et al., 1991; Chen et al., 2021; Ellis et al., 2017; Prodorutti & Frilli, 2008) have enabled pollinator dependence ratios to be calculated for raspberries (Klein et al., 2007). These studies found a reduction in fruit yield of between 10% and 70% when pollinators were excluded in comparison with open‐pollinated flowers. This range runs substantially lower than the dependence category of 40%–90% yield loss in the absence of pollinators, reported for raspberry by Klein et al. (2007), and its central point is lower than the central value of 65% used in economic analyses by Lautenbach et al. (2012) and Potts et al. (2016). This suggests that although pollinators improve raspberry yields, they are not essential for raspberry plants to produce fruit and their value may previously have been overestimated. Although most cultivars are considered self‐fertile (Keep, 1968), perhaps gaining this ability during their domestication (Jennings, 1988), the number of fruit produced and the number of seeds and drupelets within those fruits when left to self, varies between cultivars (Pinczinger et al., 2021).

To some extent, the differences in pollinator dependence estimated by these studies may reflect the true pollinator dependence of different raspberry cultivars. This highlights the need to assess the pollinator dependence in multiple cultivars, also highlighted for apple by Garratt et al. (2014), when looking to determine the pollinator dependence of raspberry crops as a whole. However, a number of other factors are known to contribute to variation in measured pollinator dependence ratio, including abiotic and biotic factors, such as soil fertility (especially nitrogen availability), temperature, water availability, and the composition of the pollinator community (e.g., see Chen et al., 2021) as well as pest levels or control (Lundin et al., 2013; Sutter & Albrecht, 2016). The extent to which these factors explain the differences in estimates of pollinator dependence in the literature is largely unknown and requires substantial additional research in each crop type, across multiple systems, to elucidate.

Methodological details, including the selected yield parameters and number of study years, are also likely to be important sources of variation in pollinator dependence ratio, as shown clearly for *Vicia faba* by Bishop et al. (2020). Rather surprisingly, to our knowledge, commercial quality and size thresholds below which fruit would not be marketable, have not previously been used to study pollinator dependence in raspberry. Unlike strawberries and apples, there is no government class specification for raspberries in the UK, instead, this is normally dictated by retailers who differ in their requirements (BerryWorld pers. Comms, December 2019).

Of the raspberry pollinator exclusion studies cited above, only Prodorutti and Frilli (2008) performed exclusion studies for more than 1 year. Pollinator community composition has been shown to vary markedly between years at the same site (Rader et al., 2012; Russo et al., 2015; Senapathi et al., 2021), and we might expect measured pollinator dependence also to vary as a result. This is because the comparison between bagged and open flowers in standard exclusion experiments measures the pollination service being provided by the pollinator community that happens to be present, in that particular ecological context. Garibaldi et al. (2011) reported that the interannual stability of pollinator‐dependent crops is lower than the stability of pollinator‐independent crops, likely due to this variability and the close relationship between pollinator species richness and plant reproductive success and yield (Albrecht et al., 2012). Multi‐year analyses are therefore essential, to determine how inter‐annual variability affects individual pollinator‐dependent crops and their resulting pollinator dependence, especially when estimating the economic value of pollinators.

Using exclusion studies over 3 years, we experimentally tested the combined effects of pollination treatment, study year, and crop cultivar on raspberry yield for two different metrics (fruit set and fruit weight), with and without accounting for marketability. We calculated the different pollinator dependence ratios and determined whether there was a pollination deficit despite highly managed pollinator input. We asked whether and how much the pollinator dependence ratio differed between years, cultivars, and yield metrics to determine whether these are likely causes of the variation in pollinator dependence found between raspberry pollination studies. We asked whether the mean pollinator dependence taken from Klein et al. (2007) and used by Lautenbach et al. (2012) and Potts et al. (2016) is representative of raspberry pollinator dependence in our study system considering this variation between years, varieties, and yield metrics. We also calculated the impact of implementing market thresholds within the yield metrics, on economic valuations of pollination service provision to commercial UK raspberry crops.

## 2 MATERIALS AND METHODS

### 2.1 Study site

The study was carried out on an 81‐ha commercial soft fruit farm near Reading, south England (51°29′32″N, 000°52′28″W) throughout the period from June to September 2019, 2020, and 2021. Two self‐compatible cultivars of red raspberry (*Rubus idaeus*); “Diamond Jubilee” and “Sapphire” were included in the study, both developed by BerryWorld and made available for growers in 2013 (BerryWorld pers. Comms, December 2022). Both cultivars were grown throughout each study period. Each experimental site was made up of one commercial field of >1.5 ha surrounded by uncropped field margins. There were small areas of semi‐natural grassland and patchy woodland on the farm and within the immediate surrounding area. Both raspberry cultivars were grown under Spanish polytunnels in drip‐fertilized and irrigated pots, each with two canes per pot. Raspberry canes are only harvested for one growing season, and 153 and 149 rented honeybee hives were in place at the farm during 2019 and 2020, respectively, equating to ~2 hives/ha of farmland, throughout the raspberry flowering season, for the purpose of crop pollination of both raspberries and strawberries. This dropped to 81 colonies in 2021, due to colony losses and relocation to other sites reducing the stocking rate to 1 hive/ha. No managed bumblebee colonies (*Bombus terrestris*) were active on the farm during the study periods in 2019 and 2020. A few colonies were still active in an adjacent field to Diamond Jubilee in 2021; however, they were at the end of their 10th week in situ when the first study flowers opened. It is therefore likely that the bees leaving these colonies were gynes and males.

### 2.2 Exclusion study pollination treatments

Crop plants were randomly selected across each field in each year; 19 in 2019 (Diamond Jubilee: *n* = 9, Sapphire: *n* = 10), 30 in 2020 (Diamond Jubilee: *n* = 10, Sapphire: *n* = 20), and 36 in 2021 (Diamond Jubilee: *n* = 16, Sapphire: *n* = 20). Canes were only used for one growing season at the study site and so new canes were selected each year for the exclusion study. Cultivars of raspberry and strawberry were also rotated between fields and so repeated sampling from the same field over multiple years was not possible. Sample size was increased in later years to provide contingency because some inflorescences were lost from the experiment in 2019 and 2020, due to accidental picking or disease. For each plant, three lateral branches with ≥7 flower buds were selected and randomly assigned to one of three treatments; insect pollination (IP), insect exclusion (IE), and insect exclusion with pollen supplementation (IES) or “hand pollination,” for 2020 and 2021 this was exactly 10 buds per treatment on each plant but for 2019 this varied between 7 and 18. Any open flowers were removed at the start of the study. The flowers assigned to the insect exclusion and insect exclusion with pollen supplementation treatments were covered by 27 × 27 cm bags made of 1 mm mesh, tied at the bottom with string to prevent pollination by insects (see Figure S1 for an image of this setup). The tops of the bags were folded and sealed with paperclips to allow them to be easily opened during hand pollination and harvesting without damaging the flowers or developing fruit. Another treatment of insect pollination with pollen supplementation (IPS) was included in 2020 and 2021 to give a maximum potential fruit production value, as this was not provided in 2019 by the insect exclusion with pollen supplementation treatment due to hand‐pollinated flowers in bags yielding significantly less fruit than open‐pollinated flowers. The flowers for this treatment were left un‐bagged to allow insect pollinators to visit and were hand pollinated with additional pollen. The pollen‐supplemented flowers were hand‐cross pollinated using a soft bristle paintbrush to transfer pollen from non‐study to study flowers of the same cultivar. Flowers were not emasculated for any of the treatments and so self‐pollination was still possible. By leaving flowers intact, our treatments replicate the current commercial yield (insect pollination), the maximum possible fruit yield (insect pollination with pollen supplementation), and the expected yield if all insect pollinators were lost, with (insect exclusion with pollen supplementation) and without (insect exclusion) human intervention. Hand‐pollinated flowers were pollinated at least twice during their receptive period (≥2 days), with the first pollination event no more than 2 days since flower opening (Andrikopoulos & Cane, 2018; Bekey, 1985). Flowers were only pollinated on dry days as pollen was hard to collect and transfer when wet.

Many Diamond Jubilee fruit were lost in 2019 to commercial harvests and so, for Sapphire, which flowered later in 2019 and for both cultivars in 2020 and 2021, bags were placed over the developing fruit for the insect‐pollinated treatments once all flowers had dropped all their petals and the tips of their stamens had turned brown and started drying. In 2021, some insect pollination with pollen supplementation fruit was lost to commercial harvests before bags could be added. Fruit picked by the harvesters was included in both fruit set analyses, identified by the presence of a receptacle, as it was assumed that they were marketable when picked.

### 2.3 Fruit collection and measurement of fruit set and quality

Fruit (entire raspberries, comprising multiple drupelets) were harvested when bright red and the fruit could be detached easily from the receptacle and counted. In 2020 and 2021, fruits were also weighed and measured (length and width) at the widest points using calipers. Fruits that were visibly infected with molds such as *Botrytis* or *Phytopthora* species, which both cause small, hardened unripe fruit, were excluded from the analysis as pollinator dependence could not be assessed. All other fruits were included in the analysis for fruit set, but only those that were classed as “marketable” by satisfying the criteria for commercial whole fruit sales were included as marketable fruit. To be counted as marketable, each fruit had to be whole (i.e., *not* missing drupelets or crumbly), without excessive bubbled drupelets (drupelets of dramatically different sizes, see Figure S2a for examples), have a length of ≥15 mm, and a weight of ≥3 g (the minimum requirement of any BerryWorld affiliated retailer; BerryWorld pers. Comms, December 2019). The pollinator dependence ratio of the crop was defined as the proportion of yield in the insect‐pollinated (IP) yield treatment that was directly attributable to insect pollinators. We calculated this for each of the following yield metrics; fruit set (%), marketable fruit set (%), fruit weight (g) of each individual fruit, and marketable fruit weight (g) of each individual fruit (excluding unmarketable fruit).

This was calculated using the following formula:

(1) $$D=\frac{{\bar{x}}_{\mathrm{IP}}-{\bar{x}}_{\mathrm{IE}}}{{\bar{x}}_{\mathrm{IP}}}$$

where *D* represents the pollinator dependence ratio, ${\bar{x}}_{\mathrm{IP}}$ is the mean yield metric (e.g., % fruit set) for insect‐pollinated flowers, and ${\bar{x}}_{\mathrm{IE}}$ is the mean yield metric (e.g., % fruit set) for insect‐excluded flowers.

Fruit set was calculated as the percentage of flowers that resulted in fruits, so buds that did not result in a flower were excluded from this analysis.

### 2.4 Economic valuation

The economic value of insect pollination to raspberry production was calculated following the bioeconomic approach of Gallai et al. (2009). This equation uses the pollinator dependence ratio (*D*), along with the quantity of commercial crop (in tonnes) produced (*Q*) and the price per tonne received at market (*P*):

(2) Economic value of insect pollination=D×Q×P

For this study, we used Defra's horticultural statistics (Defra, 2021) which gave a total national production economic value (Q×P) for 2020 (2021 data not confirmed at time of submission) multiplied by our dependence ratios for fruit set and marketable fruit set for the two cultivars and 3 years combined. Due to the differing prices per tonne for each retailer, cultivar‐specific economic valuations of pollination services to the crop were not calculated. For illustration purposes only, we used the pollinator dependence ratios calculated in this study to represent all UK‐grown commercial raspberry crops in this equation.

### 2.5 Statistical analyses

Analyses were conducted using general(ized) linear mixed models (GLMMs) in R statistical software (version 4.1.3; R Core Team, 2022) using the glmmTMB R package (v. 1.1.5; Brooks et al., 2017) and the lmerTest R package (v.3.1.3; Kuznetsova et al., 2017). Four responses were analyzed: fruit set, marketable fruit set, fruit weight, and marketable fruit weight, and each was tested against two major explanatory fixed effects: pollination treatment and year. Cultivar was also included as a fixed effect for the fruit set models as both cultivars were modeled together. Models testing fruit set and marketable fruit set had a binomial response of the number of successes (flowers that developed into fruit/marketable fruit) and failures (flowers that did not develop a fruit/developed an unmarketable fruit) per treatment branch, accounting for differences in the number of flowers per branch, and a beta‐binomial error distribution was used to account for overdispersion. Fruit weight and marketable fruit weight were normally distributed and modeled with a Gaussian error structure. Nested random effects were included in each model to account for the structure of the experimental design, shared growing conditions, and pollinator exposure, and to avoid pseudoreplication issues, with plant ID within field ID used for fruit set and marketable fruit set models, and branch ID within plant ID for fruit weight and marketable fruit weight models. Branch ID was not necessary as a random effect in the fruit set models as the response was already modeled per branch, and field ID was not included in the fruit weight model due to lack of power and very minimal impact. For per berry marketable fruit weight, the two cultivars Diamond Jubilee and Sapphire were modeled separately as the differences in fruit size and appearance are already known and are the primary reason for the cultivation of both varieties at the study farm. Maximal models were employed without simplification and acceptable model fit was assessed from residual plots. Fruit width and length were not modeled as they were both found to have a significant positive correlation with fruit weight (Corr.coeff = .895, *p* < .0001, and Corr.coeff = .855, *p* < .0001 respectively). Full details of all variables and maximal models are shown in Table 1.

**TABLE 1:** Description of the structure of each response and explanatory variable used, and the GLMM structures constructed from them.

Type	Variable	Distribution (link/offset)	Definition
Response	Fruit set	Beta‐binomial (logit)	Proportion of flowers producing a fruit (fruit/no fruit) accounting for number of study flowers on each lateral branch
	Marketable fruit set	Beta‐binomial (logit)	Proportion of flowers producing a fruit of a marketable size and quality (marketable/not marketable) accounting for number of study flowers on each lateral branch^a^
	Fruit weight	Gaussian (identity)	Mass in grams of individual fruit
	Marketable fruit weight	Gaussian (identity)	Mass in grams of individual fruit that was of marketable size and quality^a^
Explanatory	Pollination treatment	Four‐level categorical factor	Insect pollination (IP), Insect exclusion (IE), insect pollination with hand‐pollen supplementation (IPS), and insect exclusion with hand‐pollen supplementation (IES)
	Year	Three‐level categorical factor for fruit set/marketable fruit set models	2019–2021
		Two‐level categorical factor for fruit weight/marketable fruit weight models	2020–2021
	Crop cultivar	Two‐level categorical factor	Two varieties of commercial raspberry (Diamond Jubilee and Sapphire), grown in separate fields
Random	Field/Plant_ID	Six‐level categorical factor/72‐level categorical factor	Unique identifier for each study plant within each field (72 plants across 6 fields and 3 years (2019:2021))
	Plant_ID/Branch	60‐level categorical factor/four‐level categorical factor	Unique identifier for each study plant and each branch within the study plant (four branches on each of 60 plants across 2020 and 2021)
**Response**	**Model structure**		
Fruit set	Crop cultivar + Pollination treatment + Year + (1|Field/Plant_ID)		
Marketable fruit set	Crop cultivar + Pollination treatment + Year + (1|Field/Plant_ID)		
Diamond Jubilee weight	Pollination treatment + Year + (1|Plant_ID/Branch)		
Sapphire weight	Pollination treatment + Year + (1|Plant_ID/Branch)		
Diamond Jubilee marketable weight	Pollination treatment + Year + (1|Plant_ID/Branch)		
Sapphire marketable weight	Pollination treatment + Year + (1|Plant_ID/Branch)		

*Note*: The years included in each model are provided separately for the fruit set and fruit weight models as only 2 years of fruit weight data were used.

^a^ See Section 2.3 for a description of the required size and quality for marketability.

## 3 RESULTS

In total, 2456 ripe fruit were harvested from 2733 study raspberry crop flowers across both varieties, all years, and pollination treatments. About 110 of these were harvested by commercial pickers so we could not weigh or measure them, though they were included as marketable fruit in the fruit set analyses, and 385 fruits were excluded from all analyses as they were infected with *Botrytis* sp. or *Phytopthora* sp. Flowers and fruits on lateral branches damaged by humans were also removed from the study. Of the 447 unmarketable berries across all pollination treatments in 2020 and 2021, 60% were deemed unmarketable due to bubbles or crumbliness, 31% were due to both bubbles or crumbliness and being underweight (due to low drupelet numbers), and only 6% was due to being underweight without the presence of bubbles or crumbliness.

### 3.1 Fruit set

**FIGURE 1:**
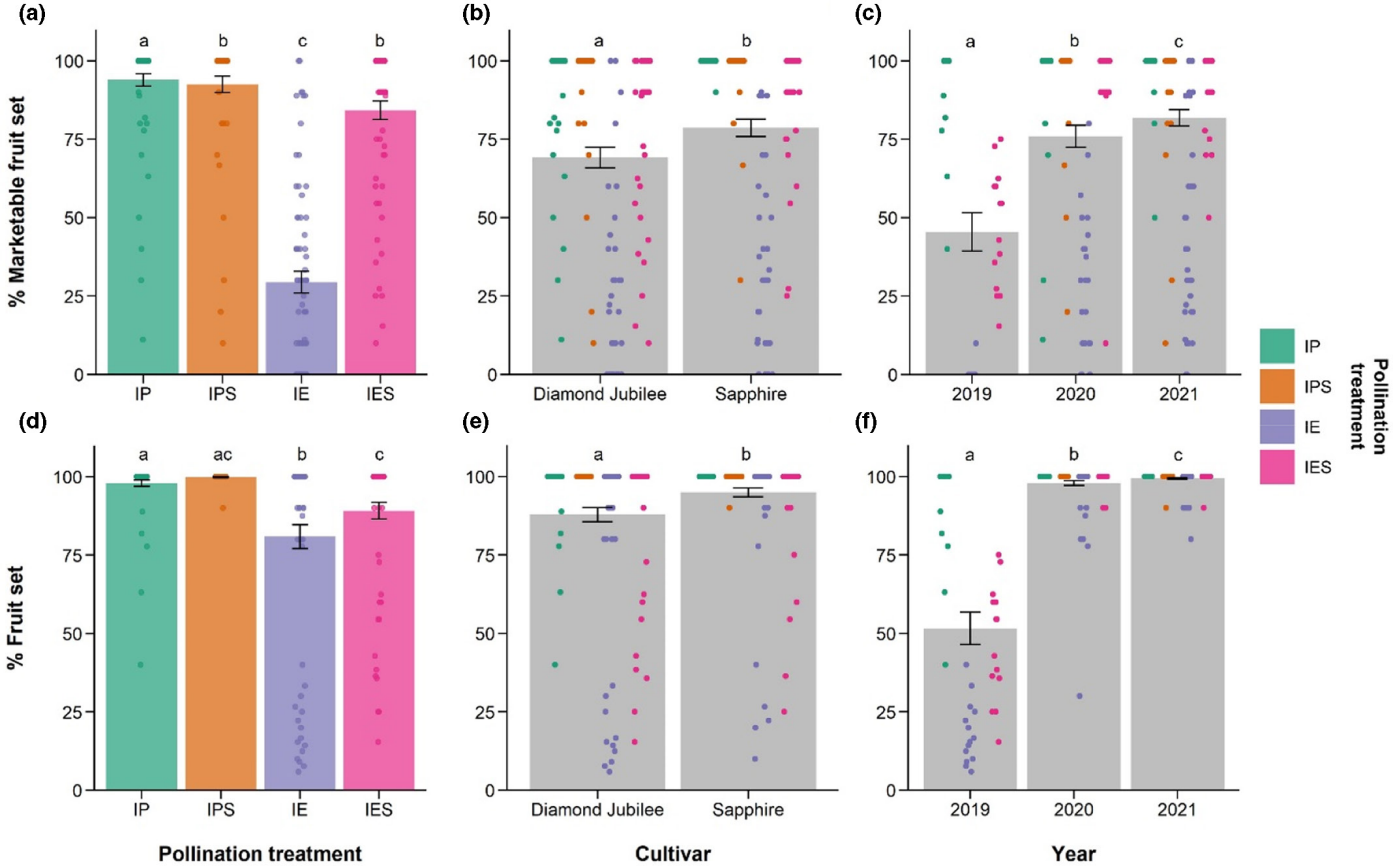
The mean percentage of study flowers that produced a marketable fruit (a–c) and a fruit whether marketable or not (d–f) under (a, d) four different pollination treatments, for (b, e) two commercial cultivars of *Rubus idaeus*; “Diamond Jubilee” and “Sapphire,” and (c, f) three consecutive study years. Pollination treatments were Insect Pollinated (IP), Insect Pollinated with hand‐pollen Supplementation (IPS), Insect Exclusion (IE), and Insect Excluded with hand‐pollen Supplementation (IES). Standard errors are shown. Different letters show significant differences between levels of each variable.

The percentage of raspberry flowers that produced marketable fruit was related to crop cultivar, year, and pollination treatment (Figure 1a–c; Table S1). Hand‐pollen supplementation of insect‐pollinated flowers (IPS: *n* = 58 branches, 92.53% ± 2.58) did not significantly increase the percentage of marketable fruit set compared with insect‐pollinated bagged branches (IP: *n* = 72, 93.93% ± 2.00; Figure 1a; *z* = −2.574, *p* = .01), in fact, marketable fruit set was significantly lower for pollen supplemented flowers, perhaps due to interference between pollen tubes. These results show that there was no pollination deficit for raspberry crop flowers in this system when producing marketable fruit. Insect‐excluded branches (IE: *n* = 72, 29.41% ± 3.48) yielded significantly less marketable fruit than insect pollinated (Figure 1a; *z* = −11.556, *p* < .0001), showing that pollinators are important for producing highly marketable yields of these cultivars of raspberry. Hand‐pollinated bagged branches (IES: *n* = 71, 84.23% ± 2.91) yielded significantly less marketable fruit than insect‐pollinated branches (Figure 1a; *z* = −4.592, *p* < .0001), indicating a negative effect of hand pollination, perhaps due to physical damage or incomplete pollination. Cultivar was a significant predictor of marketable fruit set, with Sapphire (*n* = 142 branches, 78.64% ± 2.80) yielding a significantly higher % marketable fruit set from all study branches across all treatments compared to Diamond Jubilee (*n* = 131 branches, 69.16% ± 3.27; Figure 1b; *z* = 2.173, *p* = .0298). The total marketable fruit set differed between all 3 years (Figure 1c), highlighting the variability of crop yields between years; fruit set was significantly higher in 2020 (*n* = 100 branches, 75.96% ± 3.54) and 2021 (*n* = 131 branches, 81.84% ± 2.59) than in 2019 (*n* = 42 branches, 45.49% ± 6.11; *z* = 5.200, *p* < .0001 and *z* = 6.764, *p* < .0001 respectively) and was also significantly higher in 2021 than in 2020 (*z* = 2.115, *p* = .0344; Figure 1c; Table S1).

We found similar results when using the total fruit set without the marketability threshold (Figure 1d–f; Table S1). Pollen supplementation of insect‐pollinated flowers (IPS: *n* = 58 branches, 99.83% ± 0.17) did not significantly increase the percentage of fruit set compared with insect‐pollinated branches (IP: *n* = 72, 97.93% ± 1.05; Figure 1d; *z* = −1.059, *p* = .2896). Insect‐excluded branches (IE: *n* = 72, 80.89% ± 3.84) yielded significantly less fruit than insect‐pollinated branches (Figure 1d; *z* = −9.040, *p* < .0001). Hand‐pollinated bagged branches (IES: *n* = 71, 89.13% ± 2.67) yielded significantly less fruit than insect‐pollinated branches (Figure 1d; *z* = −6.178, *p* < .0001). Cultivar was a significant predictor of fruit set, with Sapphire (*n* = 142 branches, 94.97% ± 1.43) yielding a significantly higher % fruit set from all study branches across all treatments compared with Diamond Jubilee (*n* = 131 branches, 87.85% ± 2.27; Figure 1e; *z* = 2.198, *p* = .0279). Fruit set was significantly higher in 2020 (*n* = 100 branches, 97.95% ± 0.90) and 2021 (*n* = 131 branches, 99.47% ± 0.23) than in 2019 (*n* = 42 branches, 51.63% ± 4.71; *z* = 10.067, *p* < .0001 and *z* = 9.365, *p* < .0001 respectively) and was also significantly higher in 2021 than in 2020 (*z* = 2.253, *p* = .0242; Figure 1f; Table S1). The pollinator dependence ratio for both varieties combined is shown in Table 2 and visualized in Figure 3, showing the variability in dependence ratios between years and yield criteria.

**FIGURE 2:**
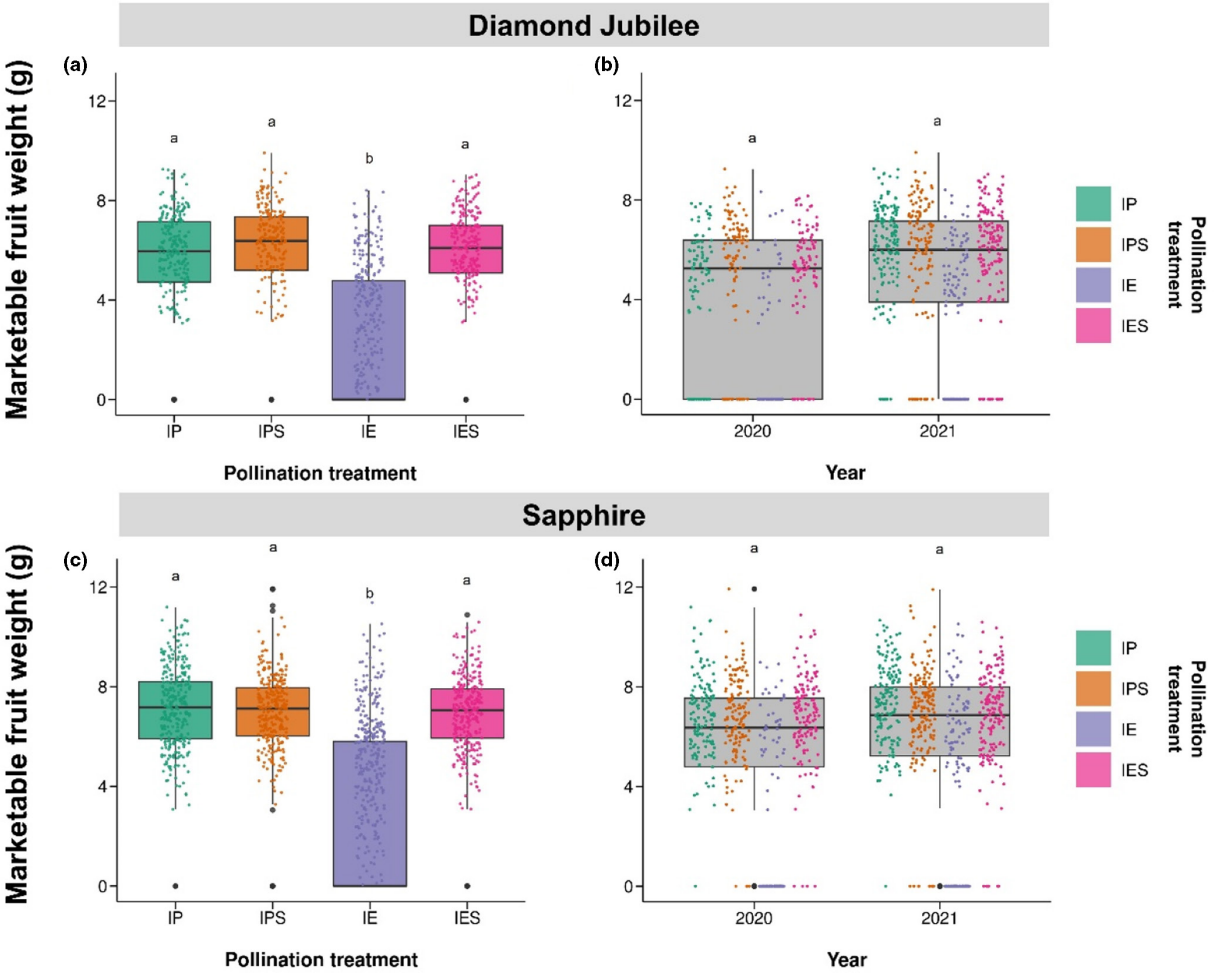
The median marketable fruit weight in grams of fruit produced by study flowers under four different pollination treatments (a and c) for two commercial cultivars of *Rubus idaeus*; “Diamond Jubilee” (a and b) and “Sapphire” (c and d) for two consecutive years; 2020, 2021. Pollination treatments were Insect Pollinated with hand‐pollen Supplementation (IPS), Insect Pollinated (IP), Insect Excluded with hand‐pollen Supplementation (IES), and Insect Exclusion (IE). IQR, minima, maxima, and outliers are shown. Outliers were <1.5*IQR from either end of the box. Different letters show significant differences between levels of each variable and each combination of variables within interactions. See text for sample sizes.

**TABLE 2:** Pollinator dependence ratios for each yield metric for both Diamond Jubilee and Sapphire over 3 years.

Cultivar	Year	Pollinator dependence ratios			
		Fruit set (%)		Fruit weight	
		Total	Marketable	Total	Marketable
**Year totals**					
Diamond Jubilee: Sapphire	2019	0.78	0.99	–	–
Diamond Jubilee: Sapphire	2020	0.07	0.72	0.30	0.60
Diamond Jubilee: Sapphire	2021	0.02	0.54	0.30	0.70
**Cultivar totals**					
Diamond Jubilee	2019:2021	0.23	0.70	–	–
Sapphire	2019:2021	0.10	0.66	–	–
Diamond Jubilee	2020:2021	–	–	0.35	0.62
Sapphire	2020:2021	–	–	0.27	0.65
**Metric totals**					
Diamond Jubilee: Sapphire	2019:2021	0.16	0.68	–	–
Diamond Jubilee: Sapphire	2020:2021	–	–	0.30	0.64

*Note*: Pollinator dependence is calculated using Equation 1. Values used for economic valuation are shown in bold.

**FIGURE 3:**
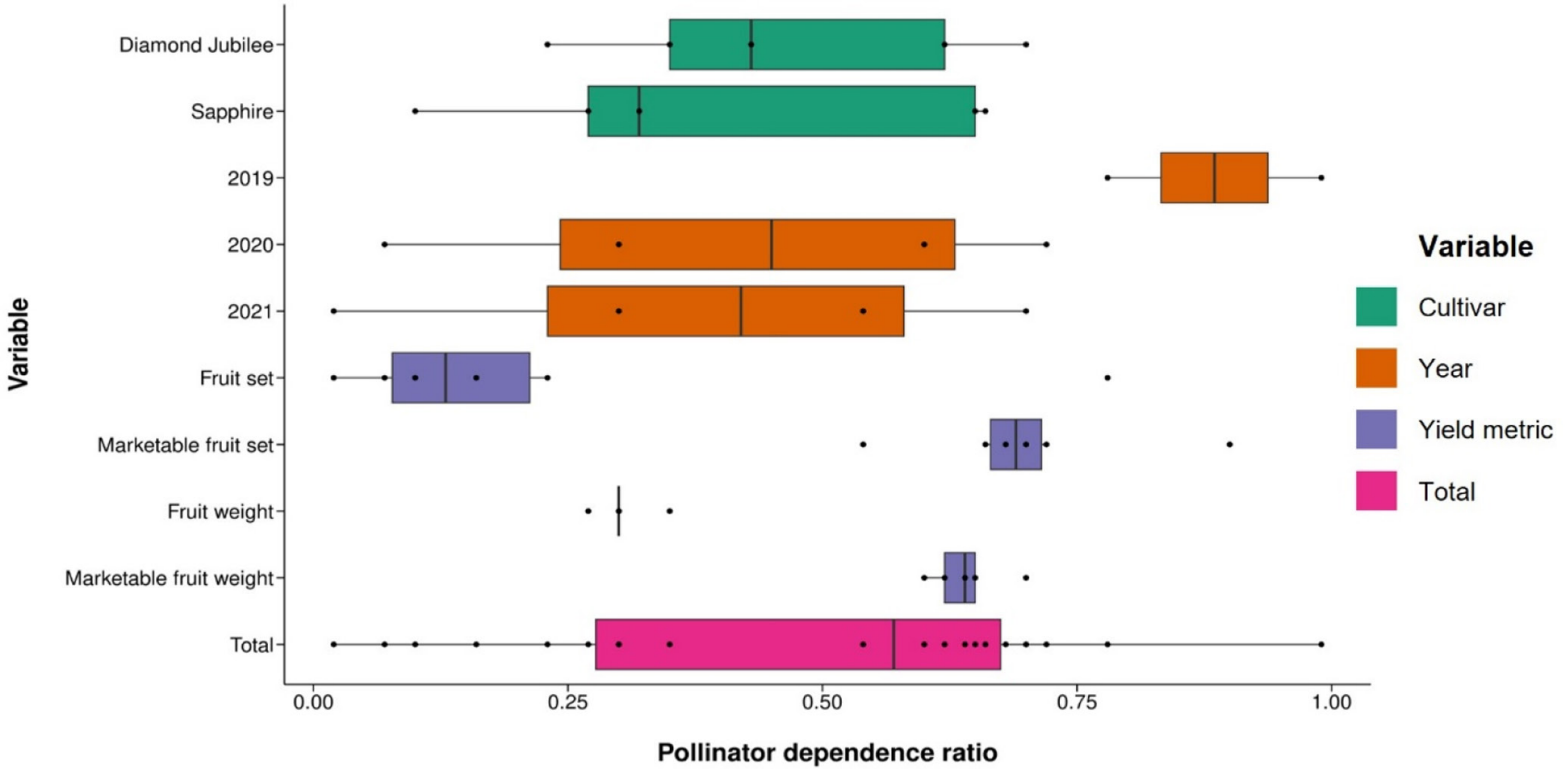
The median pollinator dependence of *Rubus idaeus* for “Diamond Jubilee” and “Sapphire” cultivars in three consecutive years (2019, 2020, and 2021) was calculated using Fruit set (%), Marketable fruit set (%), Fruit weight, and Marketable fruit weight. This is a visualization of the pollinator dependence values in Table 2. IQR, minima, maxima, and data points are shown.

### 3.2 Fruit weight

Our results for the effects of pollination treatment and year on marketable fruit weight are shown in Figure 2 and Table S2. Similar results for total fruit weight (including nonmarketable fruit) are provided in Figure S3 and Table S3. Pollen supplementation of insect‐pollinated flowers did not significantly increase marketable fruit weight compared with fruit from insect‐pollinated flowers for either Diamond Jubilee (IP: *n* = 154 fruit from 26 branches, 5.41 ± 0.15, IPS: *n* = 222 fruit from 26 branches, 5.62 g ± 0.17; *t* = 0.610, *p* = .544) or Sapphire (IP: *n* = 206 fruit from 32 branches, 7.11 g ± 0.10, IPS: *n* = 313 fruit from 32 branches, 6.80 g ± 0.11; *t* = −1.027, *p* = .307) suggesting that marketable fruit weight was not pollen limited in either variety. The pollinator dependence ratio was calculated using measurements in this study. We have also shown that the pollinator dependence ratio using fruit weight marketable fruit weight for both cultivars is shown in Table 2 in comparison with the other yield metrics including nonmarketable fruit to show how accounting for marketable quality when measuring the % of crop yield in grams affected the resulting pollinator dependence ratio. There was no significant year effect for either cultivar (Diamond Jubilee: *F* = 3.313, *p* = .08, Sapphire: *F* = 1.828, *p* = .187; Figure 2b,d).

### 3.3 Economic valuation of pollination to UK raspberry production

The economic value of insect pollination to raspberry production was calculated using Equation 2, multiplying the UK total national production economic value for raspberry (Defra, 2021) by the overall pollinator dependence ratios for % fruit set (0.16), % marketable fruit set (0.68), fruit weight (0.30), and marketable fruit weight (0.64) for all years and cultivars combined. This valued the benefit of insect pollinators to UK raspberry production in 2020 at £21.3 million using the % of fruit set and this value was quadrupled to £90.6 million when market thresholds were taken into account using the % of marketable fruit set. For fruit weight, this was £39.9 million, more than doubling to £85.3 million using marketable fruit weight.

## 4 DISCUSSION

The importance of insect pollination to UK raspberry production is evidenced by the average reduction in marketable fruit set of 68.2% (Diamond Jubilee: 70.4%, Sapphire: 66.4%, *D* = 0.68) across our two varieties and three study years (Table 2) when pollinators were excluded. This is similar to the central dependence value for raspberry by Klein et al. (2007). The benefit provided by pollinators in this system was valued much lower using the % of fruit set (16.34%, *D* = 0.16), as expected due to the self‐compatibility of cultivated *R. idaeus*. This yield metric included low‐quality fruit that would not reach the market as fresh raspberries, and therefore have no economic value for commercial producers in our system, negating its usefulness in pollinator dependence ratios for commercial crops.

When our pollinator dependence ratios were used to estimate the economic value of insect pollination to UK raspberry production, the value dramatically changed depending on which yield metric was used. The value to UK raspberry production in 2020 using our total marketable fruit set dependence ratio was ~£90 million compared with £21.3 million using a fruit set. Smith et al. (2011) give the pollinator dependence of UK raspberries as 0.45, quoting a 2007 monetary value of pollinators to UK raspberries as £39 million per annum. Since 2007, the economic value of UK raspberry production has increased by almost 50% from £90.7 million in 2007 to £133.3 million in 2020. Insect pollination of raspberry crops was therefore worth ~£60 million in 2020 using the same 0.45 dependence ratio. The £30 million per annum difference in pollinator value reflects the different estimates of the dependence ratio (0.68 in this study vs. 0.45 in Smith et al., 2011). The economic value of our study cultivars is also likely to differ due to their differing pollinator dependence values. Calculating cultivar‐specific pollinator dependence values prevents over or underestimating the value of pollinators to individual cultivars using averaged crop pollinator dependence (Garratt et al., 2014) and informs the cultivar‐specific importance of effective pollination management. Where cultivar‐specific production quantities and prices are available, these should be used to provide the economic value of pollinators to each cultivar. If economic valuations continue to be used to highlight the risks associated with pollinator declines (Silva et al., 2021), and to argue the necessity of pollinator conservation efforts (but see Kleijn et al., 2015), more accurate pollinator dependence ratios are required, using commercially relevant yield metrics.

Measuring fruit set alone does not accurately capture the benefits of insect pollinators to crop yield, because quality criteria relating to fruit weight, size, and appearance can exclude some fruit from the market (BerryWorld pers. Comms, December 2019). Marketable fruit weight and marketable fruit set together provide a more complete picture of the benefit of insect pollinators to crop yield where yield is paid for by weight rather than units. In this study, bubbles or crumbliness (Figure S2a) were the main cause of berries being considered unmarketable, rather than berries being underweight. The large disparity between the fruit set and marketable fruit set pollinator dependence ratios in this study is therefore mainly due to the positive effect of pollinators on the uniformity and number of seeds/drupelets and the resulting fruit cohesion rather than their benefit to fruit weight. Not accounting for the benefit pollinators provide to fruit aesthetics in this study therefore substantially underestimates the commercial value of their pollination service provision. Where pollination levels affect crop size, weight, or esthetics, as demonstrated here and also in pollination studies for apples (Garratt et al., 2014), cotton, and sesame (Stein et al., 2017), strawberries, oilseed rape, and buckwheat (Bartomeus et al., 2014; Klatt et al., 2014), fruit set alone does not capture the true pollinator dependence of commercial crop production and the benefit pollinators have on crop yields.

Hand pollination was included as a treatment in the first year (2019) as this was thought to represent the maximum fruit set as in other crops (Garratt et al., 2014). However, in that year, hand pollination yielded significantly fewer marketable fruit than insect‐pollinated flowers. The insect pollinated with hand supplementation treatment was therefore added in 2020 to represent the maximum potential fruit set to determine whether there was a pollination deficit. Having both treatments helps separate the effect of the bag from the effect of preventing cross‐pollination. Both bagged treatments yielded significantly fewer marketable fruit in 2019 than in either of the other study years. The extremely low yields for insect‐excluded flowers in 2019, which lead to the higher dependence ratio values, are therefore not solely due to lack of pollination. The UK experienced a heatwave in July 2019 (max daily temp of ≥35°C for 5 days recorded on the farm, reaching 40°C on one of these days) during the flowering period of the study raspberry crops. The effect of these high temperatures on raspberry yields has not been previously studied, but pollen viability and seed set in flowering plants can be negatively impacted by high temperatures (Descamps et al., 2018; Devasirvatham et al., 2012; Hedhly, 2011) and these effects can be mediated by insect cross‐pollination (Bishop et al., 2016). Mesh, like that used for our exclusion bags, has been shown to increase the temperature underneath or inside it by 0.7°C (Alaphilippe et al., 2016). Our mesh size was even finer than this and so could have increased the temperature inside by a larger margin, potentially enhancing the negative effect of pollinator exclusion on the fruit set and inhibiting insect‐excluded flowers from self‐fertilizing as well as causing a reduction in the hand‐pollinated fruit set compared with 2020 and 2021. The unexpected reduction in fruit set in hand‐pollinated flowers relative to insect‐pollinated flowers in 2019 could be explained either by a temperature‐related effect of the mesh bag or by the mediative effects of pollinators on open‐pollinated flowers that were also potentially damaged by excessive heat (Bishop et al., 2016). This potential interaction between temperature and pollination treatment is rarely accounted for and so we retained the results from this year as the low yields in 2019 represent a genuine source of variation in results between sites, and years, for a given crop. Temperature sensors could be placed inside exclusion bags to monitor temperatures to assess this effect in future studies. Climate variation has been found to explain a third of global crop yield variability for the globally important crops wheat, maize, rice, and soybean (Ray et al., 2015), while fruit set and fruit weight of strawberries (Menzel, 2019) and tomatoes (Vijayakumar et al., 2021) has been found to decrease with temperature increases. Considering the effect of temperature on crop yields and pollinator dependence is thus important, especially in the context of climate change, with increasingly unpredictable weather conditions expected (IPCC, 2022).

The mean pollinator dependence ratio using % fruit set (*D*_*i*_ = 0.16) for this study was similar to the 20% mean yield reduction reported by Prodorutti and Frilli (2008), for ripe fruit set measured over 2 years, despite our study having a much larger range in pollinator dependence using this yield metric (0.02–0.78 compared with 0.14–0.26). This shows the importance of multi‐year studies for providing representative mean pollinator dependence ratios despite interannual variation in fruit yields. For the marketable fruit set, our pollinator dependence ratio varied less between years but was still highly variable (0.54–0.99). This interannual variation in dependence demonstrates substantial stochastic uncertainty in estimates of pollinator dependence, even for a specific cultivar in a single location, and suggests that multi‐year analyses are necessary to establish estimates with realistic uncertainty ranges. Taken as a whole, our data set provides strong evidence of interannual variation in pollinator dependence estimates, which can have dramatic effects on subsequent crop pollination service economic valuations, as shown in this study. The causes of this interannual variation could be due to the differing pollinator community, environmental effects such as weather, temperature, pest levels, or differing fertilizer and water inputs (Chen et al., 2022) or limitations or it could be due to plant fertility and genetics. These potential causes of variation in crop yield exist within commercial farms and thus measuring pollinator dependence in multiple years at the same site can help capture the pollinator dependence range of that crop. Future exclusion studies should endeavor to collect data in as many years as possible to allow this uncertainty to be accounted for and, ideally, measured.

The total variation in pollinator dependence ratio, dependent on yield metric, year, and cultivar, in this study (0.02–0.99) was greater than the between‐study variation for previous raspberry pollination studies (10%–70% yield reduction in the absence of pollinators). This suggests that between‐study variation is likely to be at least in part, explained by differences in yield metric, study year, and cultivar. Using a multi‐year study and a more appropriate yield metric, we have validated the *Rubus ideaus* pollinator dependence value used by Lautenbach et al. (2012) and Potts et al. (2016).

## 5 CONCLUSION

We have shown that two varieties of commercially produced raspberry in the UK (Diamond Jubilee and Sapphire) are pollinator dependent using four different yield metrics over three study years. However, the strength of this dependence is highly sensitive to the cultivar, year, yield metric used, and the environmental conditions of the study, as well as the criteria used to decide which fruit are included in “yield” measurements. Where exclusion studies are used to calculate the economic value of pollination services using dependence ratios, we strongly recommend that studies are conducted over multiple years (three or more) to generate a range of uncertainty, and that commercial quality criterion linked to actual market value are incorporated into the calculations of dependence.

## AUTHOR CONTRIBUTIONS

**Imogen C. Ryan:** Conceptualization (equal); data curation (lead); formal analysis (lead); investigation (lead); methodology (lead); project administration (equal); visualization (lead); writing – original draft (lead). **Lynn V. Dicks:** Conceptualization (equal); funding acquisition (lead); methodology (supporting); project administration (equal); supervision (lead); writing – review and editing (lead). **Jack D. Shutt:** formal analysis (supporting); supervision (supporting); writing – review and editing (supporting)

## CONFLICT OF INTEREST STATEMENT

The authors declare that they have no conflict of interest.

## Supporting information


Data S1.

## Data Availability

Data are available to view and download from the Environmental Information Data Centre here https://doi.org/10.5285/de5b4f33‐f679‐4798‐8daf‐51a314e78204 R code for the analysis performed, and plots created for this paper are available using this github link https://github.com/imogen‐constance/Raspberry_pollinator_dependence.
