# Supplementary material for: The importance of multi‐year studies and commercial yield metrics in measuring pollinator dependence ratios: A case study in UK raspberries Rubus idaeus L
Source: Ecol Evol. 2023 May 7;13(5):e10044. doi: 10.1002/ece3.10044 (PMC10164645; doi:10.1002/ece3.10044)
Supplement: Supplementary file 1 — Data S1. [file ECE3-13-e10044-s001.docx]

**Table S1.** Results of Generalised Linear Mixed Models (GLMMs) with beta binomial error distributions for the response of **fruit set** for (a) marketable fruit and (b) total fruit.

| Variable | Estimate | Std. error | z ratio | P value |
| --- | --- | --- | --- | --- |
| a) |  |  |  |  |
| (Intercept) | 1.5279 | 0.3887 | 3.931 | **<0.001** |
| Cultivar:Sapphire | 0.6051 | 0.2784 | 2.173 | **0.0298** |
| Year:2020 | -2.1501 | 0.4135 | 5.200 | **<0.001** |
| Year:2021 | 2.8115 | 0.4157 | 6.764 | **<0.001** |
| Treatment:IE | -5.0481 | 0.4369 | -11.556 | **<0.001** |
| Treatment:IES | -1.7074 | 0.3718 | -4.592 | **<0.001** |
| b) |  |  |  |  |
| (Intercept) | 2.1636 | 0.4031 | 5.368 | **<0.001** |
| Cultivar:Sapphire | 0.7677 | 0.3492 | 2.198 | **0.0279** |
| Year:2020 | 4.3782 | 0.4349 | 10.0682 | **<0.001** |
| Year:2021 | 5.6151 | 0.5996 | 9.365 | **<0.001** |
| Treatment:IE | -4.0211 | 0.4448 | -9.040 | **<0.001** |
| Treatment:IES | -2.5462 | 0.4122 | -6.178 | **<0.001** |
| Treatment:IPS | -1.1759 | 1.1102 | -1.059 | 0.2896 |

**Table S2.** Results of General Linear Mixed Models (GLMMs) of **marketable fruit weight** with Gaussian errors for (a) Diamond Jubilee and (b) Sapphire

| Variable | Estimate | Std. error | t value | P |
| --- | --- | --- | --- | --- |
| a) |  |  |  |  |
| (Intercept) | 4.8001 | 0.4793 | 10.017 | **<0.001** |
| Year:2021 | 0.9414 | 0.5172 | 1.820 | 0.081 |
| Treatment:IE | -3.3696 | 0.4183 | -8.054 | **<0.001** |
| Treatment:IES | 0.2357 | 0.4164 | 0.566 | 0.573 |
| Treatment:IPS | 0.2560 | 0.4197 | 0.610 | 0.543 |
| b) |  |  |  |  |
| (Intercept) | 6.8261 | 0.3275 | 20.843 | **<0.001** |
| Year:2021 | 0.5129 | 0.3801 | 1.352 | 0.186 |
| Treatment:IE | -4.5700 | 0.2841 | -16.088 | **<0.001** |
| Treatment:IES | -0.3004 | 0.2863 | -1.049 | 0.297 |
| Treatment:IPS | -0.2911 | 0.2834 | -1.027 | 0.307 |

**Table S3.** Results of Generalised Linear Models GLMMs of **fruit weight** with gaussian errors for (a) Diamond Jubilee and (b) Sapphire

| Variable | Estimate | Std. error | t value | P |
| --- | --- | --- | --- | --- |
| a) |  |  |  |  |
| (Intercept) | 5.7783 | 0.3316 | 17.423 | **<0.001** |
| Year:2021 | 0.3078 | 0.3735 | 0.824 | 0.418 |
| Treatment:IE | -2.1234 | 0.2550 | -8.327 | **<0.001** |
| Treatment:IES | 0.0978 | 0.2536 | 0.386 | 0.701 |
| Treatment:IPS | 0.3459 | 0.3560 | 1.351 | 0.181 |
| b) |  |  |  |  |
| (Intercept) | 6.8770 | 0.2699 | 25.476 | **<0.001** |
| Year:2021 | 0.4425 | 0.3218 | 1.375 | 0.179 |
| Treatment:IE | -1.8898 | 0.2174 | -8.691 | **<0.001** |
| Treatment:IES | -0.1561 | 0.2192 | -0.712 | 0.478 |
| Treatment:IPS | -0.0531 | 0.2170 | -0.245 | 0.807 |


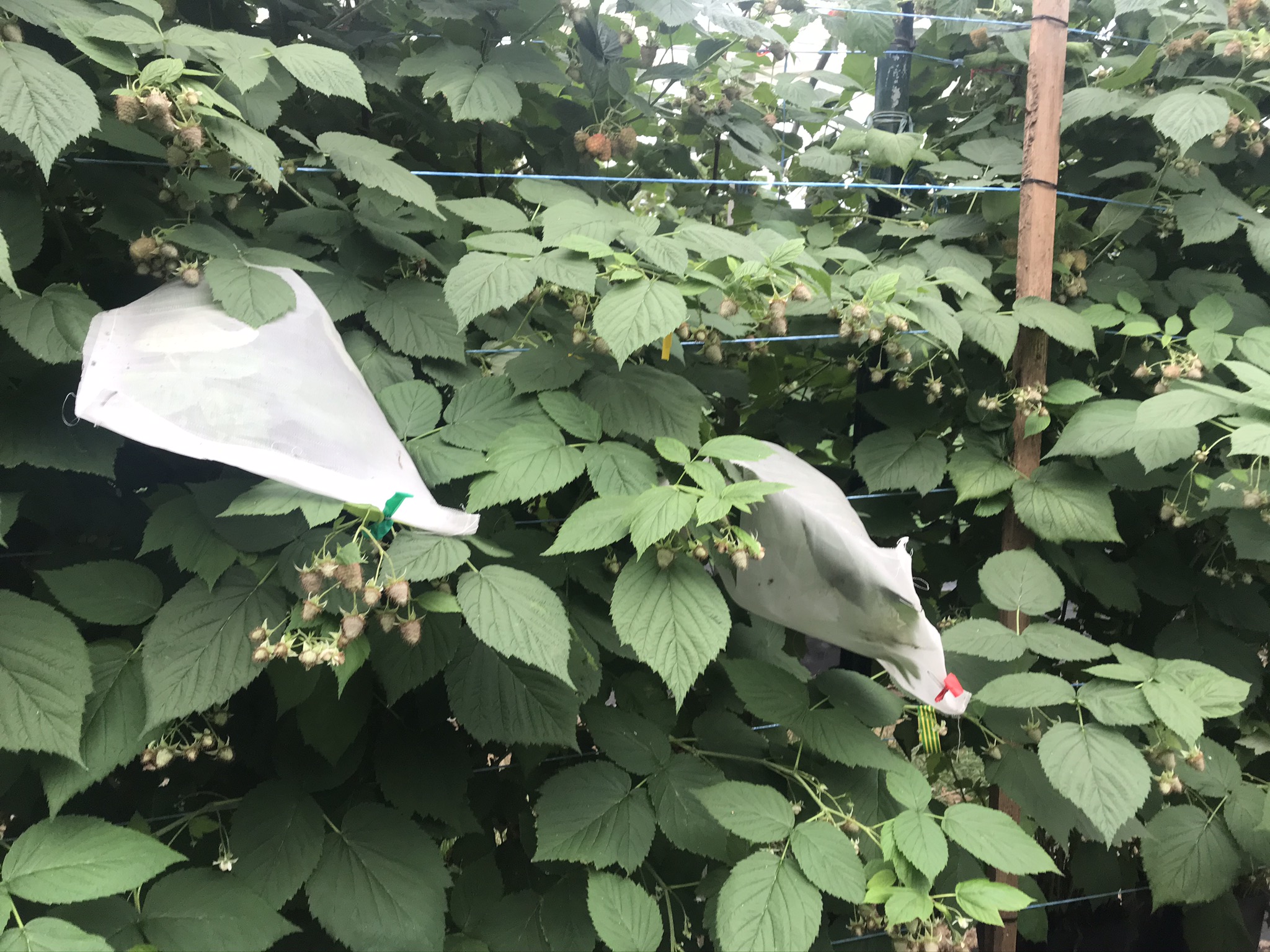

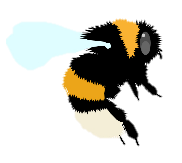


**Insect pollination (IP)**


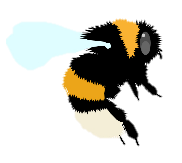


**Insect pollination + pollen supplementation (IPS)**


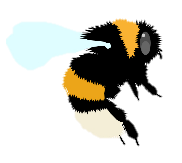

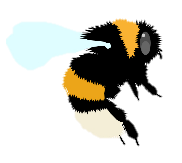


**Insect exclusion (IE)**


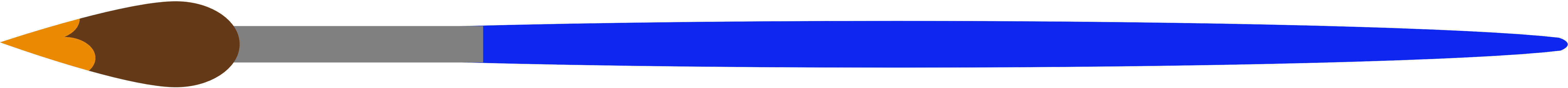

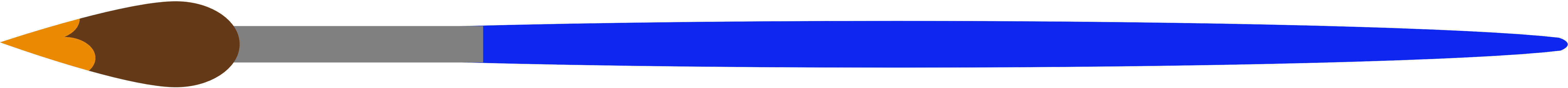


**Insect exclusion + pollen supplementation (IES)**

**Figure S1**. Example of exclusion study set up with four branches on the same raspberry cane assigned to each of the four pollination treatments and marked with the corresponding colour of tape. Insect exclusion with pollen supplementation (circled in pink) and insect exclusion (circled in lilac) branches have sealed mesh bags to exclude insects from accessing the flowers.


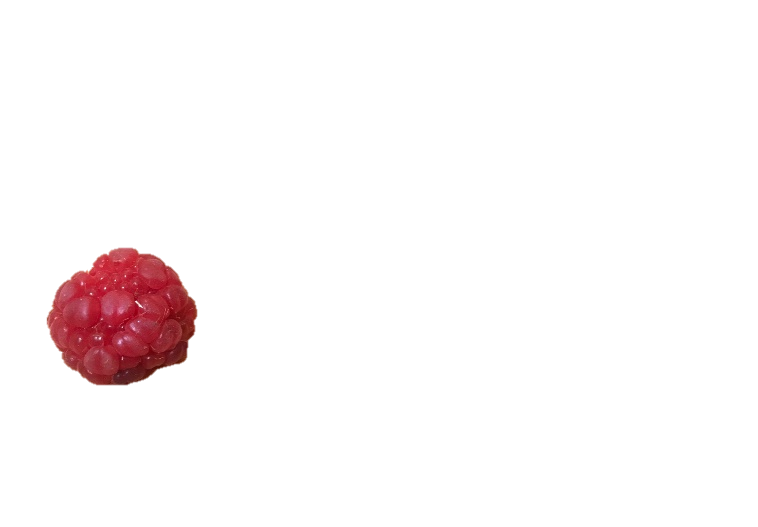

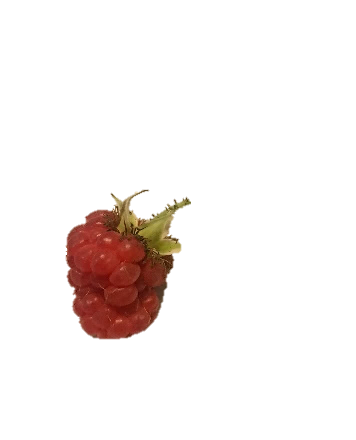

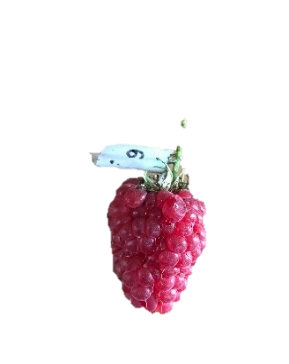

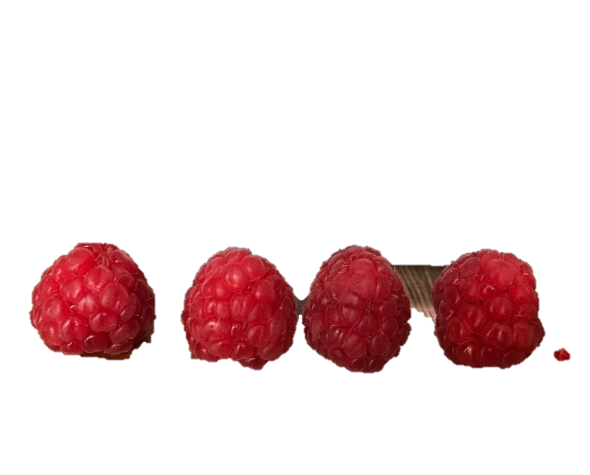

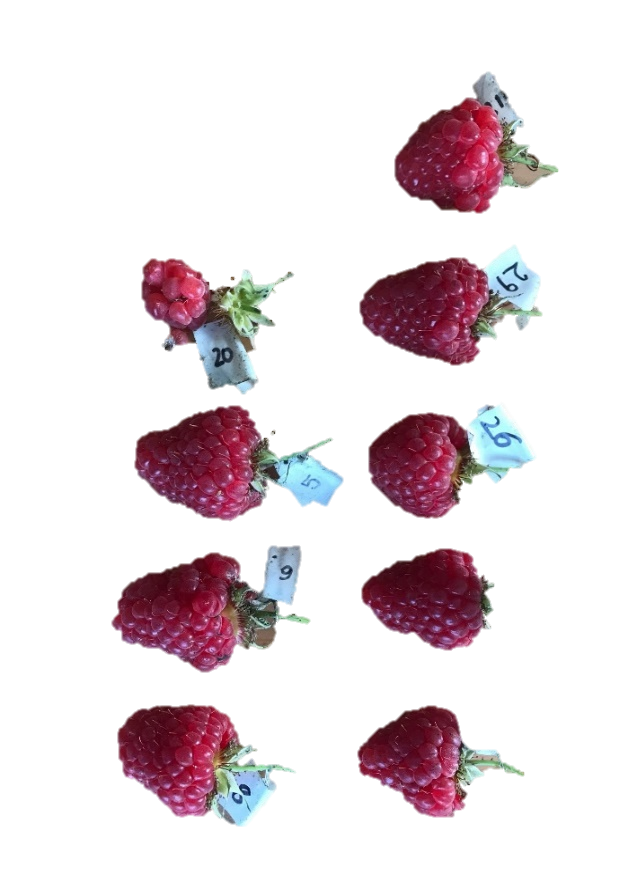

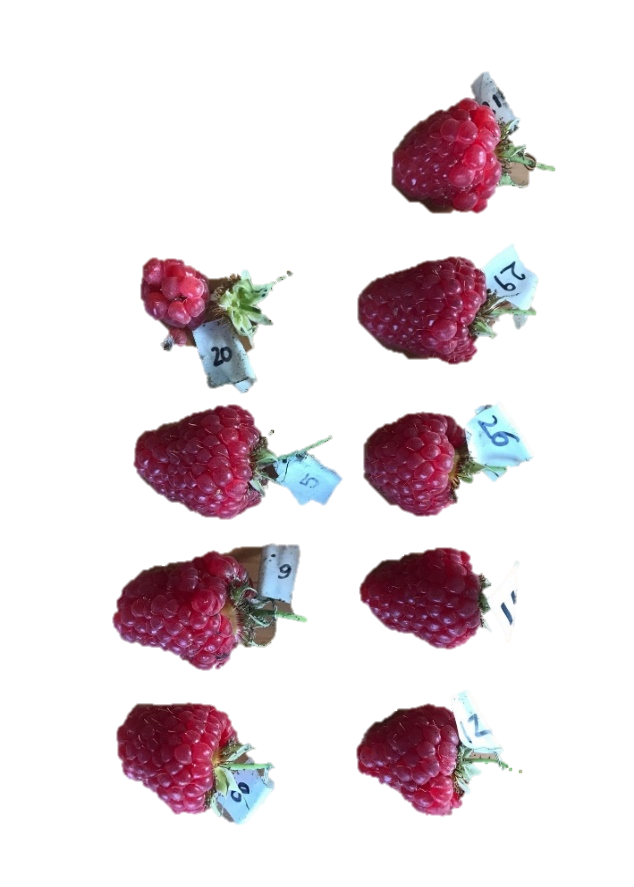

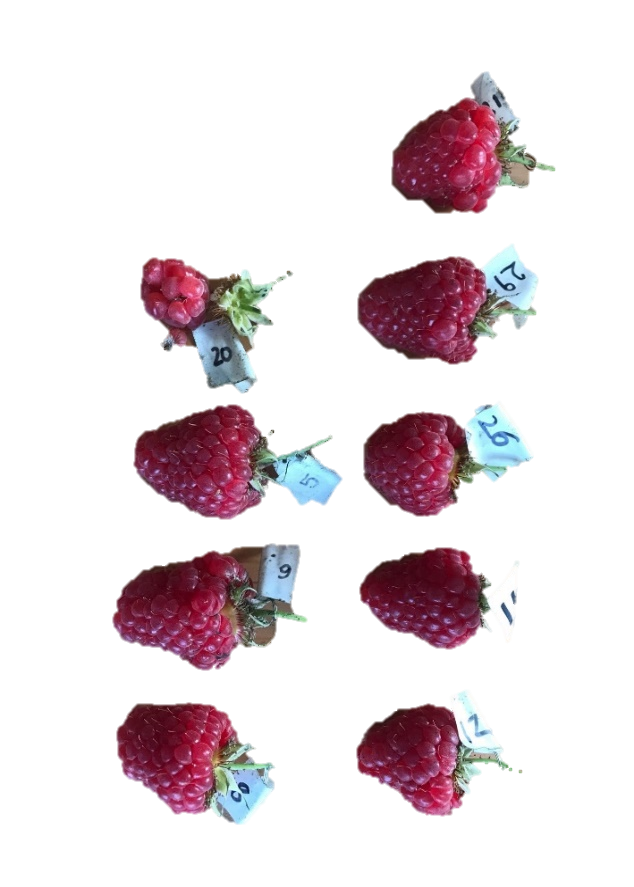

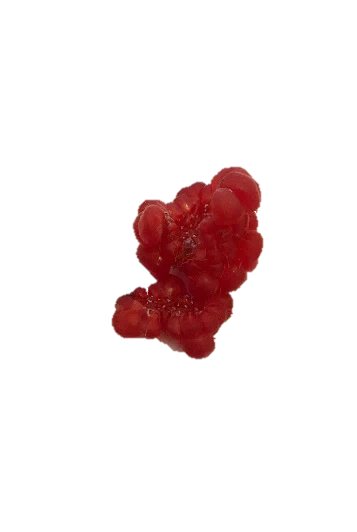

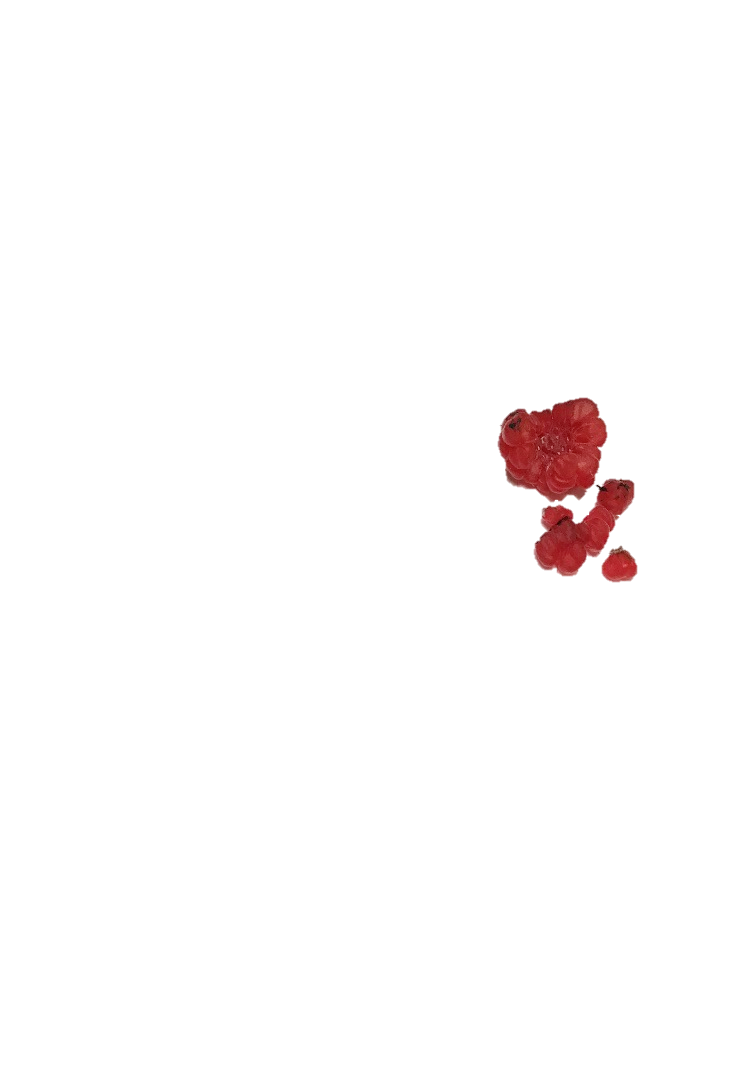


a

b

c

**Figure S2.** Examples of a) unmarketable bubbled fruits, b) unmarketable crumbly fruits and c) marketable whole fruits


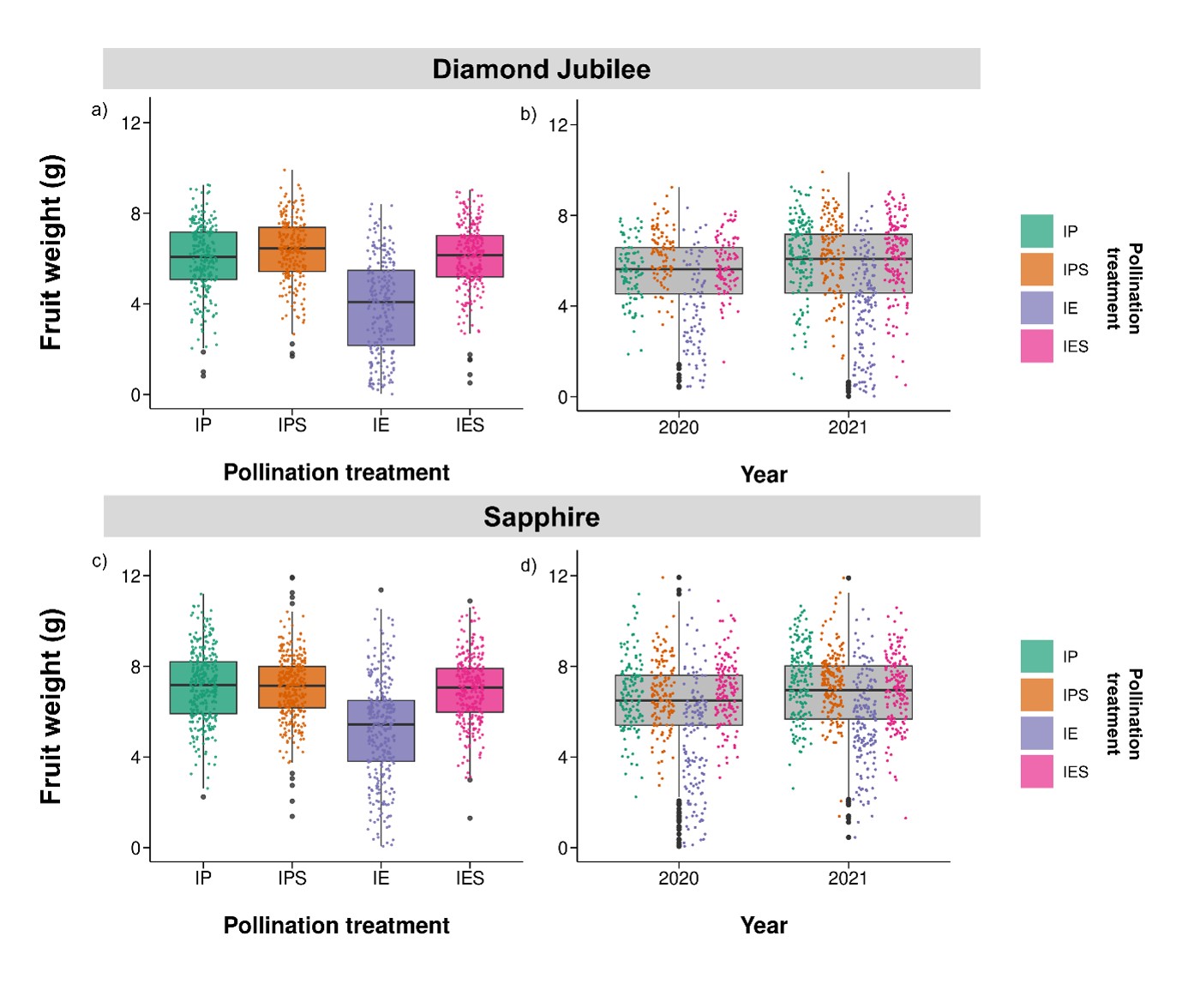


**Figure S3.** The median marketable fruit weight in grams of fruit produced by study flowers under four different pollination treatments (a&c) for two commercial cultivars of *Rubus idaeus*; ‘Diamond Jubilee’ (a&b) and ‘Sapphire’ (c&d) for two consecutive years; 2020, 2021. Pollination treatments were Insect Pollinated with hand pollen Supplementation (IPS), Insect Pollinated (IP), Insect Excluded with hand pollen Supplementation (IES) and Insect Exclusion (IE). IQR, minima, maxima and outliers are shown. Outliers were <1.5*IQR from either end of the box. Different letters show significant differences between levels of each variable and each combination of variables within interactions. See text for sample sizes.
